# Supplementary material for: P-TRAP: a Panicle Trait Phenotyping tool
Source: BMC Plant Biol. 2013 Aug 29;13:122. doi: 10.1186/1471-2229-13-122 (PMC3848748; doi:10.1186/1471-2229-13-122)
Supplement: Additional file 7 — P-TRAP source code. The Netbeans project that contains the source code of the application. [file 1471-2229-13-122-S7.zip › javadoc.zip!/javadoc/org-basmati-workspace/org/basmati/workspace/package-summary.html]

org.basmati.workspace (WorkSpace)


JavaScript is disabled on your browser.


- Package
- Class
- Tree
- Deprecated
- Index
- Help

*org.basmati.workspace 1.0*

- Prev Package
- Next Package

- Frames
- No Frames

- All Classes

# Package org.basmati.workspace

- Class Summary

  | Class | Description |
  | --- | --- |
  |  |  |
  | --- | --- |
  | CollectMasterReportAction | The action handler when the user click on the collect report button. |
  | ColoredImageAction | The handler of of the "colored image" button action. |
  | Drawer | Superimpose graph and particles on a given image |
  | EditandViewGrainFileActionDataObject | The data object implementation to represent the grain result file. |
  | EditResultFileAction | The handler of the view/edit result file action. |
  | EditViewGrains | The handle of the view/edit grain action. |
  | ExpressRunAction | The handler of the run express command in the tool bar. |
  | FindGrainsAction | The handler of finding all grains in all image in the selected project. |
  | FindGrainsSingleImageAction | The handler of detecting the grains in a single image action from the context menu. |
  | FindGrainTraitsAction | The handler of the find grain traits action. |
  | FindGrainTraitsSingleImage | The handler of the find grain trait action for a single image from the context menu. |
  | FindSkeletonAction | The handler of finding the structure of all images in the selected project. |
  | FindStructureSingleImageAction | The handler of finding the structure in the selected image |
  | GrainsFinder | Grains finder finds the grains in both a single image and a set of images. |
  | GrainTraitsFinder | The grain's traits finder class works for both a single image and a set of images. |
  | GraphEditorTopComponent | Top component which displays the source images. |
  | GraphUI | The GraphScene which shows the structure as a graph. |
  | ImageScaler | The scaler of the input images in the selected project. |
  | ImageTypeAction | The handler of the image background type option. |
  | ImageViewScene | The Scene that shows the input images. |
  | ImportImages | Handel the import image action. |
  | OpenImageRice | The Handler when the user opens a single image in the image viewer. |
  | ParticleScene | The `GraphScene` which shows the grains and allows the user to edit them. |
  | ParticlesEditorTopComponent | Top component which displays the grains and allows editing them. |
  | ProcessedRiceImageFileDataObject | A `MultiDataObject` class that represents the processed image. |
  | ProjectUIManager | The project handler class. |
  | ReportGenerator | The Reports generator. |
  | ResultProducer | It saver of all result files. |
  | ResultsEditorTopComponent | Top component which displays the results of the structure detection. |
  | ScaleImage | The handler of the images scaling action. |
  | ScaleSelectorPanel | The dialog of scaling the image. |
  | ScalingPanel | The panel of the scaling dialog |
  | StructureFinder | This class detects the structure of the panicle either in a single or multiple images. |
  | VeiwParticlesAnchors | The handler of toggling the anchor points of the grains ON and OFF. |
- Enum Summary

  | Enum | Description |
  | --- | --- |
  |  |  |
  | --- | --- |
  | GraphUI.calcType | This is the type of the calculation required on the graph |

- Package
- Class
- Tree
- Deprecated
- Index
- Help

*org.basmati.workspace 1.0*

- Prev Package
- Next Package

- Frames
- No Frames

- All Classes
